# T-Cell-Specific Membrane Antigens in the Mexican Axolotl (Urodele Amphibian)

**DOI:** 10.1155/1992/76201

**Published:** 1992

**Authors:** Fabienne Kerfourn, Francoise Guillet, Jacques Charlemagne, Annick Tournefier

**Affiliations:** 1 Laboratoire d'Immunologie Comparée Université Pierre et Marie Curie CNRS URA 1135 9 Quai Saint-Bernard Paris 75005 France; 2 Laboratoire d’Immunologie Comparée Université de Bourgogne Faculté des Sciences 6 Boulevard Gabriel Dijon 21004 France

**Keywords:** Urodele amphibian, T lymphocyte markers, polyclonal antibodies, 38-kD antigens

## Abstract

Comparative analysis of SDS-PAGE patterns of axolotl spleen cells membrane detergent lysates showed important discrepancies between control and thymectomized animals. Among these, a 38-kD protein band, which appeared as a major protein in controls, was not or poorly expressed after thymectomy. A rabbit antiserum (L12) raised against the 38-kD eluted band labeled in indirect immunofluorescence 80-86% of thymocytes and 40-46% of mIg_–_ lymphoid cells in the spleen. The anti-38-kD antibodies stained in Western blotting two antigenically related polypeptides of 38- and 36-kD on splenocyte membrane lysates. Two-dimensional NEPHGE-PAGE analysis indicated that the anti-38-kD antibodies reacted in the spleen with several gathered spots in the 7.8–8.2 pI range, corresponding to 38–36-kD microheterogeneous polypeptides. Most of these spots are not further expressed in thymectomized animals. These results support evidence that the 38-kD surface antigens can be considered as specific surface markers of the axolotl thymus-derived lymphocytes.


Developmental Immunology, 1992, Vol. 2, pp. 237–248
Reprints available directly from the publisher
Photocopying permitted by license only

© 1992 Harwood Academic Publishers GmbH
Printed in the United Kingdom

### T-Cell-Specific Membrane Antigens in the Mexican Axolotl (Urodele Amphibian)

FABIENNE KERFOURN,*† FRANCOISE GUILLET,† JACQUES CHARLEMAGNE,† and ANNICK TOURNEFIER‡

†*Laboratoire d'Immunologie Comparée, Université Pierre et Marie Curie, CNRS URA 1135, 9 Quai Saint-Bernard, 75005 Paris, France*

‡*Laboratoire d'Immunologie Comparée, Université de Bourgogne, Faculté des Sciences, 6 Boulevard Gabriel, 21004 Dijon, France*

Comparative analysis of SDS-PAGE patterns of axolotl spleen cells membrane detergent lysates showed important discrepancies between control and thymectomized animals. Among these, a 38-kD protein band, which appeared as a major protein in controls, was not or poorly expressed after thymectomy. A rabbit antiserum (L12) raised against the 38-kD eluted band labeled in indirect immunofluorescence 80–86% of thymocytes and 40–46% of $mIg^-$ lymphoid cells in the spleen. The anti-38-kD antibodies stained in Western blotting two antigenically related polypeptides of 38- and 36-kD on splenocyte membrane lysates. Two-dimensional NEPHGE-PAGE analysis indicated that the anti-38-kD antibodies reacted in the spleen with several gathered spots in the 7.8–8.2 pI range, corresponding to 38–36-kD microheterogeneous polypeptides. Most of these spots are not further expressed in thymectomized animals. These results support evidence that the 38-kD surface antigens can be considered as specific surface markers of the axolotl thymus-derived lymphocytes.

KEYWORDS: Urodele amphibian, T lymphocyte markers, polyclonal antibodies, 38-kD antigens.

### INTRODUCTION

The characterization of lymphocyte surface molecules is the subject of extensive studies and considerable progress has been made in defining T- and B-cell subpopulation markers in mammalian vertebrates over the last decade. On the contrary, the phylogenic study of lymphocyte populations in lower vertebrates underlines the scarcity of lymphocyte markers in fish, amphibians, and reptiles. Although comparative immunologists have described cellular as well as humoral responses in fish and amphibians, very little is known about their lymphocyte subpopulations. However, all the data already published clearly demonstrate a functional lymphocyte heterogeneity (reviews in Du Pasquier, 1989; Charlemagne, 1990). Allograft rejection and its in vitro correlate, the mixed lymphocyte reaction, have been described in fish and in amphibians. Moreover, amphibian and fish lymphocytes can undergo rather vigorous in vitro responses to classic mammalian B- and T-cell mitogens. In amphibians, humoral responses to antigenic stimulation are mediated through different immunoglobulin isotypes and the result of a "T-B"-like cooperation, at least in anurans. Early thymectomy abrogates the transplantation reaction and influences the antibody response, indicating clearly the significant role of the T lymphocyte compartment in immune responses of these lower vertebrates. The first molecules of the immune system to be characterized in ectothermic vertebrates were immunoglobulins (Ig) and anti-Ig reagents were developed (Ching and Wedgwood, 1967; Houdayer and Fougereau, 1972; Du Pasquier and Haimowitch, 1976; Litman, 1976; Litman and Marchalonis, 1982; Tomonaga and Kobayashi, 1985; Chardin et al., 1987). The B lymphocyte subpopulation was then defined as membrane immunoglobulin-positive ($mIg^+$) lymphocytes (Nagata and Katagiri, 1978; Lobb and Clem, 1982; Secombes et al., 1983; Miller et al., 1985; Tournefier et al., 1988a, 1988b). On the other hand, reagents defining T-cell subpopulations have been much more difficult to obtain. Many monoclonal antibodies (mAb) produced are directed against carbohydrate determinants shared between different cell lineages, probably

*Corresponding author.

because of the phylogenic distance between fish, amphibians, and mice.

The only T lymphocyte marker detected in fish was described by Miller et al., 1987. This membrane antigen is part of a multimolecular complex formed by 70-, 110- and 150-kD polypeptides, but is not restricted to the T-cell lineage. In anuran amphibians, a T-cell-specific marker has been described in *Xenopus*. This 120-kD (XTLA-1) antigen is present on 90–95% of thymocytes and 30% of splenic leucocytes. Early larval thymectomy abrogates the appearance of cells bearing this XTLA-1 determinant (Nagata, 1985, 1988).

In urodele amphibians and more precisely in the neotenic and primitive axolotl (*Ambystoma mexicanum*), one mAb has been raised (mAb 34.38.6) defining a group of 65–72-kD polypeptides present on hemopoietic stem cells and expressed throughout the T lymphocyte differentiation lineage, but also on myeloid cells (Tournefier and Guillet, 1987; Tournefier et al., 1988b). In axolotl, as in all lower vertebrates, specific T lymphocyte markers had to be defined to differentiate the T lymphocyte subpopulation among non-mIg$^+$ cells. Our attempts to define axolotl membrane antigens by cross reactions using mAb directed against bird and mammalian T lymphocyte markers were always negative. Therefore, we developed an original strategy to increase the probability of producing useful reagents. The present study describes this strategy and the characterization of 38-kD membrane antigens specific and restricted to the axolotl T lymphocyte subpopulation.

### RESULTS

### Lymphocyte Membrane Proteins of Normal and Thymectomized Axolotls

Crude membrane lysates of splenic lymphocytes were routinely analyzed by electrophoresis on 12.5% SDS-PAGE. The electrophoretic patterns of membrane proteins were fairly identical from one animal to the other at the adult stage between 14 and 24 months. Some polypeptides in the 78–70-, 44–38-kD range appeared highly expressed in control animals after Coomassie blue staining (Fig. 1, lanes 1–3). The same membrane lysates prepared from thymectomized (TX) axolotls showed strong discrepancies of electrophoretic patterns compared to controls (Fig. 1, lanes 4 and 5). Some molecules were overexpressed and others were absent. Such differences can be explained by the disappearance of thymus-derived cells, but also by some more indirect effect of thymectomy on the relative proportion of spleen leucocyte subpopulations (Charlemagne, 1983, and unpublished observations). A 38-kD protein, which appeared as a major band in control animals, seemed to be absent in the TX electrophoretic patterns. In axolotl, larval thymectomy depletes 45–50% of the peripheral lymphocyte compartment. The absence of the 38-kD molecule and the depletion of mature T cells induced by thymectomy could very well be coincidental, but could also aim the 38-kD molecule as a membrane protein specifically expressed by the T-cell subpopulation.

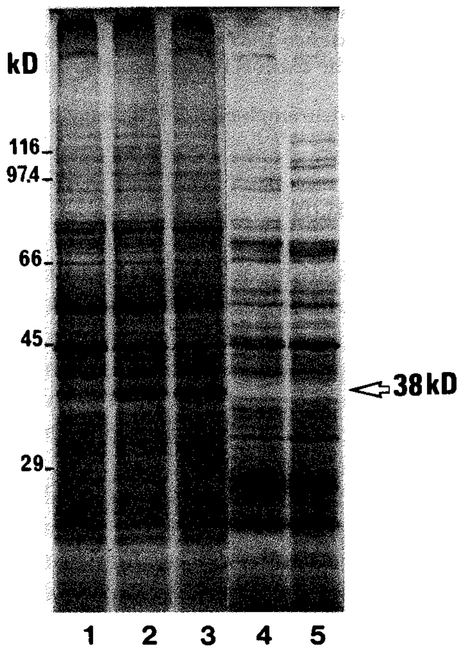

FIGURE 1. Electrophoretic analysis of axolotl splenic lymphocyte membrane proteins on 12.5% SDS polyacrylamide gel after Coomassie blue staining. Solubilized lymphocyte membrane extracts of normal (lanes 1–3) and thymectomized animals (lanes 4 and 5). Molecular weight markers indicated in kD on the left are $\beta$-galactosidase (116), phosphorylase b (97.4), albumin bovine (66), ovalbumin (45), and carbonic anhydrase (29). Arrow indicates the position of the 38-kD membrane polypeptide. This protein is abundant on splenocytes of 14–18-month-old axolotls (lanes 2 and 3), but seems not expressed on splenocytes of 14-month-old axolotls after thymectomy (lanes 4 and 5). A natural decrease of the 38-kD polypeptide expression is observed in older (24-month-old) animals (lane 1).

Therefore, this 38-kD molecule was chosen as a potential T-cell marker, electroeluted, and used as antigen for repeated immunizations in order to obtain rabbit polyclonal antibodies (L12). This L12 antiserum was further used to define by flow cytometry the tissue distribution of the 38-kD molecule and to characterize its molecular properties by Western blotting.

### Tissue Distribution of the 38-kD Molecule

The L12 purified polyclonal antibodies raised against the 38-kD molecule were tested at 4–8 $\mu$g/ml on thymic, peripheral blood, and splenic lymphocyte suspensions by indirect immunofluorescence. The observations were made by optic microscopy under a fluorescence microscope and by flow cytometry; they are summarized in Table 1. The preimmune L12-0 antiserum labeled less than 1% lymphocytes in the thymus and less than 2% in the spleen. Indirect immunofluorescence analysis showed that L12 antibodies reacted with 80–86% thymocytes and 40–46% splenocytes. Optic microscopy and flow cytometry analysis gave roughly the same results. The respective percentages of labeling were similar when L12 mono-38 was used instead of L12 antibodies.

Splenic lymphocytes were examined by two-color immunofluorescence and flow cytometry for reactivity with L12 antibodies and mAb 33.101.2. A typical experiment is illustrated in Fig. 2. L12 stained 36.8% of cells, 19.4% were labeled with mAb 33.101.2, 36.4% were negative, and 7.2% were double stained. When, on the same batch of splenic cells, L12 mono-38 was used instead of L12 antibodies, the number of lymphocytes stained by the monospecific antibody raised to 43.5% and the number of negative cells fell to 29.2%. The other percentages remained unchanged. In this experiment, the small discrepancies observed in double fluorescence between percentages of fluorescence obtained with L12 and L12 mono-38 (36.8% versus 43.5% on splenic lymphocytes) can be explained because L12 mono-38 antibodies were first adsorbed and then concentrated after elution, which may have had for results, first, selecting antibodies with a strong affinity for the 38-kD antigen, and, second, staining lymphocytes having very few 38-kD molecules on their surface. Consequently, these lymphocytes were included in the positive cell number by the very sensitive flow cytometry technique. In correlation, the percentage of double-negative cells is diminishing in the same proportions (36.4% versus 29.2%). However, when the mean percentages of splenic lymphocyte subpopulations ($L12^+Ig^-$ and $L12^+Ig^+$, respectively) were calculated on several experiments, no significant differences were observed with L12 and L12 mono-38 antibodies (Table 2). These results indicated that $L12^+$ and $mIg^+$ lymphocyte populations were mainly mutually exclusive.

TABLE 1
A Comparison of Fluorescence-Positive Thymus, Spleen, and Blood-Cell Populations as Determined by Fluorescence Microscopy and Flow Cytometry[a]

| | | Thymus | Spleen | Blood |
|---|---|---|---|---|
| Optic microscopy | L12-0 | 0.4±0.49 | 1.6±0.80 | ND[b] |
| | L12 | 95.0±0.71 | 43.9±3.45 | 42.67±1.25 |
| | L12 mono-38 | 95.0±2.18 | 45.0±3.98 | ND[b] |
| Flow cytometry | L12-0 | 0.90±0.45 | 1.90±0.30 | ND[b] |
| | L12 | 83.25±2.77 | 43.10±3.35 | 40.0±2.35 |
| | L12 mono-38 | 85.20±3.00 | 43.47±4.02 | ND[b] |

[a]Indirect immunofluoresence analysis was done as described in Materials and Methods. Cells were stained with L12-0: rabbit preimmune serum; L12: rabbit anti-38-kD antibodies; L12 mono-38: rabbit anti-38-kD antibodies eluted from the electroblotted 38-kD molecule. Numbers represent the mean percentages (±SD, calculated on at least three independent experiments) of cells observed by optic microscopy or flow cytometry.

[b]ND: not determined.

### Immunochemical Characterization of L12 Reactive Molecules

Membrane lysates of thymocytes and splenic lymphocytes were submitted to monodimensional SDS-PAGE, electroblotted, and selectively stained by L12 antibodies (Fig. 3). Under standard reducing conditions (2% SDS containing 5% 2ME for 3 min at 100 °C), L12 stained a major protein of 38 kD on thymocyte and two major polypeptides of 38 and 36 kD on splenocyte lysates. In these precise conditions, some other polypeptides, all of higher molecular masses are labeled (Fig. 3, lanes 2 and 4). As the rabbit L12 had been immunized exclusively with the 38-kD electroeluted molecule, the detection of higher molecular weight polypeptides was a problem. In view of these results, lymphocytes were incubated with iodoacetamide before and during detergent solubilization and a more drastic

It was of particular interest to note that this different conditioning of the membrane lysate samples had for result the exclusive revelation by L12 of the 38- and 36-kD polypeptides on splenocytes without any more higher molecular weight polypeptides (Fig. 3, lane 5). The preimmune L12-0 serum did not show any reactivity for membrane proteins in Western blotting (Fig. 3, lanes 1 and 3). Strong reducing conditions as well as iodoacetamide treatment were systematically used for further experiments.

To ensure that the antigen recognized by L12 antibodies on live cells by immunofluorescence is identical to the 38–36-kD antigen recognized by the same antiserum in Western blotting, we absorbed the L12 antiserum successively on two batches of splenic lymphocytes for 1 hr at 4 °C (v/v) and used this absorbed antiserum in Western blotting on splenic lymphocytes lysates. After one absorbtion, L12 antibodies still weakly reveal the 38-kD band on splenocytes (Fig. 3, lane 7). No reactivities were noticed when L12 antibodies were absorbed twice in the same conditions (Fig. 3, lane 8).

To ascertain the precise antigenic relationship between the splenic 38- and 36-kD molecules revealed by L12 antibodies, we decided to isolate the fraction of L12 antibodies specifically directed to the 38-kD protein. These L12 mono-38 antibodies behave like L12 unfractioned antibodies and in Western blotting under reducing conditions stained only the 38-kD protein on thymocytes and the 38-kD, as well as the 36-kD, molecules on splenocytes (Fig. 4, lanes 1 and 2). Under nonreducing conditions, the mobility and intensity of the 38-kD molecule remains unaffected (Fig. 4, lanes 3 and 4). However, a significant drop of the 36-kD intensity was observed in the spleen and a new molecule of high molecular weight (118 kD) appeared to be labeled (Fig. 4, lane 3).

Thymic and splenic membrane lysates of normal axolotls were submitted to two-dimensional NEPHGE-PAGE and L12 reactive molecules were revealed after immunoblotting. It was important

TABLE 2
Double Immunofluorescence Staining of Axolotl Splenic Lymphocyte Populations with L12 and mAb 33.101.2 Antibodies[a]

| | $L12^+Ig^-$ | $L12^-Ig^+$ | $L12^+Ig^+$ | $L12^-Ig^-$ |
|---|---|---|---|---|
| L12-0 | 0.75±0.15 | 30.62±4.26 | 0.13±0.05 | 68.48±4.08 |
| L12 | 37.97±2.16 | 20.03±3.74 | 9.63±2.70 | 32.17±4.83 |
| L12 m-38 | 36.80±6.70 | 23.05±4.25 | 8.50±0.20 | 31.50±2.30 |

[a]Double immunofluoresence analysis was done as described in Materials and Methods. Cells were stained with mAb 33.101.2, specific for axolotl Ig and, alternatively, with L12-0, L12 or L12 m-38 (L12 mono-38 antibodies, see Table 1). Results are expressed in percentages (±SD) of labeled cells.

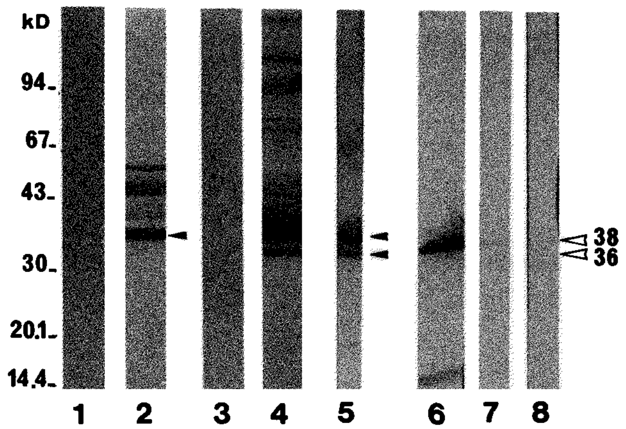

FIGURE 3. Immunoblots of thymocytes and splenic lymphocytes membrane proteins. After SDS-PAGE (12.5%) under reducing conditions, proteins were electroblotted onto nitrocellulose and transfers were revealed using L12 antibodies. Lanes 1 and 2: Membrane lysates from thymocytes. Lane 3–8: Membrane lysates from splenocytes. Cells were pretreated with iodoacetamide (lanes 5–8) or without (lanes 1–4). Immunoblots were revealed with the preimmune L12-0 serum (lanes 1 and 3), with L12 antibodies (lanes 2, 4–6), with L12 antibodies absorbed once (lane 7) or twice (lane 8) on axolotl splenocytes. Arrows indicate position of the 38- and 36-kD polypeptides. Molecular weight markers are indicated on the left.

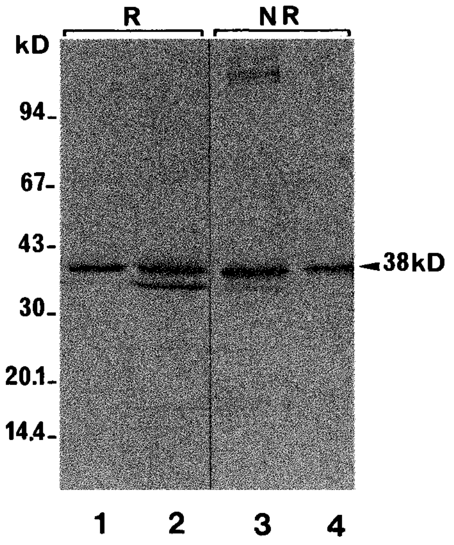

FIGURE 4. Immunoblots of thymocytes and splenic lymphocytes membrane lysate proteins under reducing (R) (lanes 1 and 2) and nonreducing (NR) conditions (lanes 3 and 4). Thymocytes (lanes 1 and 4) and splenocytes (lanes 2 and 3) membranes. Immunoblots were revealed with L12 mono-38 antibodies.

to notice that the L12 antiserum labeled a clearly limited area of spots (Figs. 5-3 and 5-4) among the large amount of polypeptides that was detected on thymocytes and splenocytes by silver nitrate staining (Figs. 5-1 and 5-2). On spleen lysates, L12 antibodies stained horizontal series of several spots in the 7.8–8.2 pI range, corresponding to 38–36-kD microheterogeneous polypeptides (Fig. 6-2). Interestingly, on spleen lysates of thymectomized axolotls, the L12 antiserum revealed only three spots, in the 38–36-kD range, and the labeling intensity was extremely weak

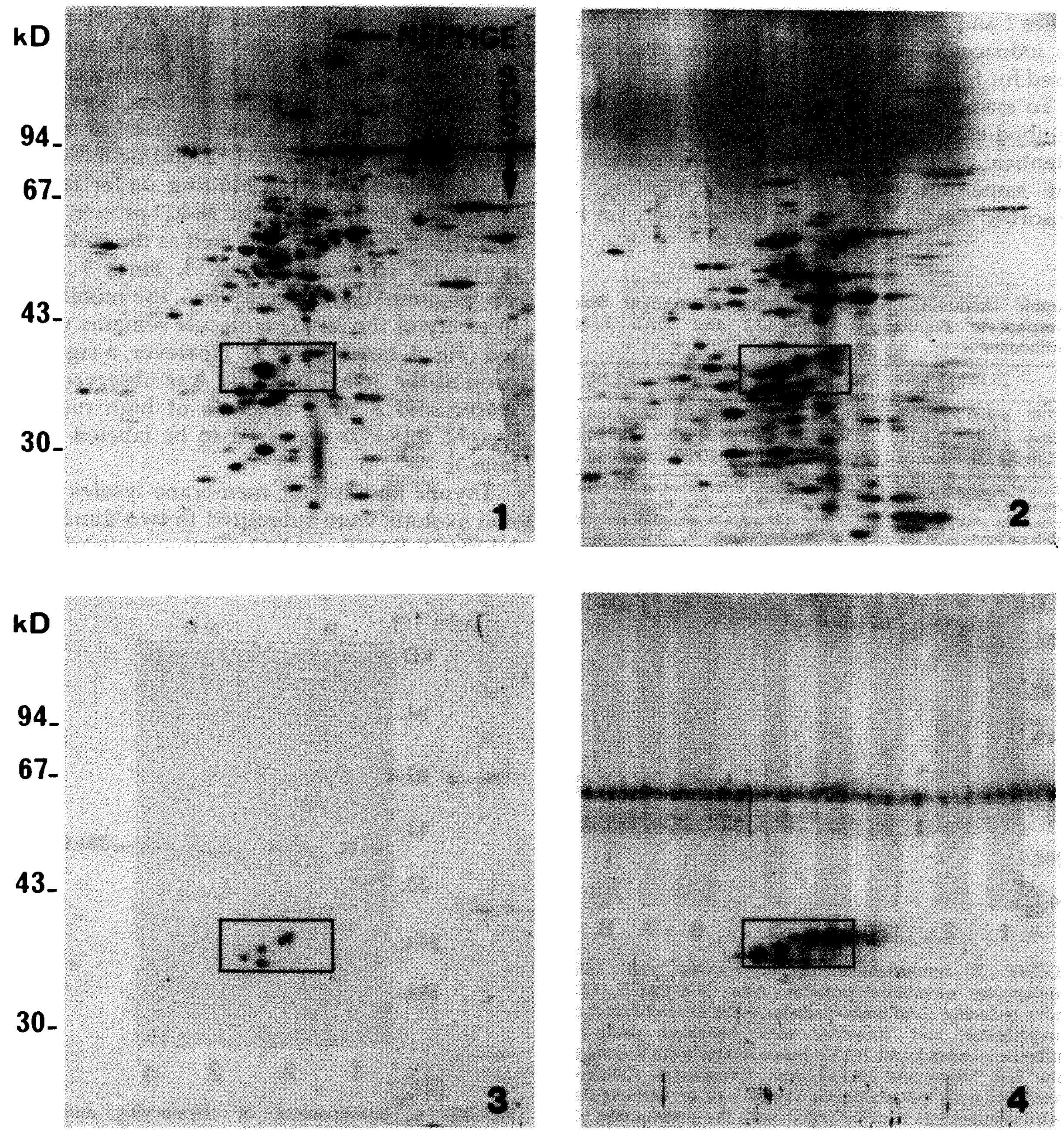

FIGURE 5. Two-dimensional analysis of lymphocyte membrane proteins in NEPHGE conditions. Thymus (1) and spleen (2) cell membrane lysate pattern (silver nitrate staining) of 15-month-old axolotls. Immunoblots of thymocytes (3) and splenocytes (4) revealed with L12 antibodies. Small square in (1) and (2) means the area of corresponding spots revealed in immunoblots (3) and (4).

(Fig. 6-3). It should be noticed that the same amounts of membrane lysate were loaded through this 2D analysis of both control and thymectomized animals and that the revelation procedure was carried in parallel with identical conditions. These results corroborated our first observation of the disappearance of the 38-kD molecule in the spleen of thymectomized animals.

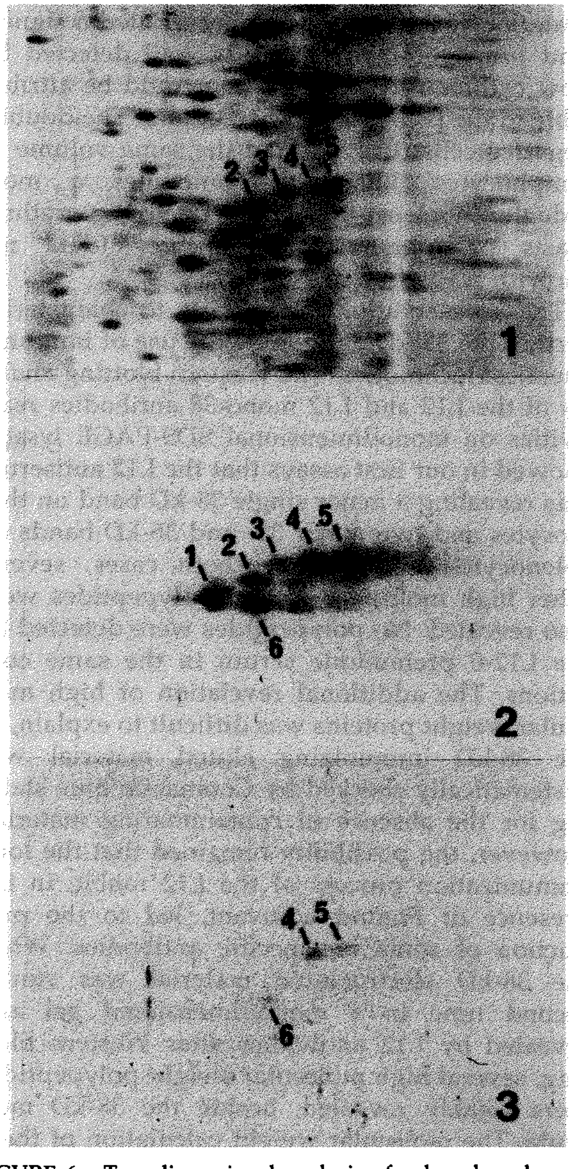

FIGURE 6. Two-dimensional analysis of spleen lymphocyte membrane polypeptides. Effect of thymectomy. The area corresponding to the proteins boxed in Fig. 5 is enlarged. (1): Protein pattern visualized by silver nitrate staining. (2) and (3): Immunoblots of splenocytes from normal (2) and thymectomized (3) axolotls revealed by L12 antibodies. Major corresponding spots are numbered. Notice the faint expression of proteins recognized by L12 antibodies in the spleen of thymectomized axolotls.

### Protein Deglycosylation

Removal of N-linked sugars from the electroeluted and isolated 38-kD protein by endo-F treatment after different enzyme concentrations (0.1 to 0.25 U) and different incubation times (2 to 18 hr) was maximal with 0.25 U of endo-F and 6 hr of incubation. This treatment yielded two major bands of 38-kD and 36-kD and a minor band of 32-kD (Fig. 7, lane 2). The same treatment applied during 18 hr gave identical results that assess for the complete hydrolysis of complex and high mannose glycans (Fig. 7, lane 3). Complete deglycosylation of axolotl Ig was previously obtained with endo-F treatment (Chardin et al., 1987). Western blotting and staining with L12 of the 38-kD deglycosylated polypeptides revealed that the remaining nonglycosylated 38-kD (endo-F insensitive) produce was the only labeled protein. The 36-kD and 32-kD deglycosylated polypeptides were not stained by L12 antibodies (Fig. 7, lane 4).

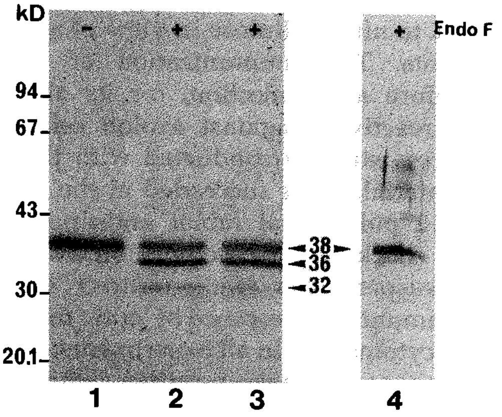

FIGURE 7. Deglycosylation of the 38-kD polypeptide. The 38-kD protein was electroeluted from splenic lymphocyte membrane lysates separated by 12.5% SDS-PAGE, and then incubated at 37 °C for 6 or 18 hr (lanes 2 and 3), with (+) or without (–) endo-F. Coomassie blue staining (lanes 1–3). The endo-F treated material (lane 2) was transferred onto nitrocellulose and revealed by Western blotting with L12 antiserum. The remaining nonglycosylated 38-kD (endo-F insensitive) product is labeled; the deglycosylated 36- and 32-kD polypeptides are not stained by L12 (lane 4).

### DISCUSSION

We reported in the axolotl the presence of 38-kD lymphocyte membrane antigenic structures that we characterized by a polyclonal rabbit antiserum (L12). An extensive analysis was performed using flow cytometry, mono- and two-dimensional electrophoresis followed by Western blotting. The results of these assays provide convincing evidence that these membrane molecules are specific of a T lymphocyte population sensitive to thymectomy.

The first part of this work aimed at producing a polyclonal rabbit antiserum specific of a 38-kD molecule present in lymphocyte membrane extracts of normal axolotl and absent of electrophoretic patterns of splenic lymphocytes in thymectomized animals. Saponin permeabilized axolotl lymphocytes were lysed in NP-40/DOC or digitonin buffer and centrifuged to discard the nuclei and the cytoskeletal debris. These lysates were not at this point considered by the authors as pure membrane extracts, but as a basic material to isolate the 38-kD protein from a monodimensional SDS-PAGE gel. The 38-kD band was electroeluted and a sample of this eluate was sytematically checked by a second run in monodimensional SDS-PAGE stained with Coomassie blue dye, before being used as antigen or blotted to nitrocellulose in view of absorbtion experiments. The immunization of a rabbit, tested before any injection, for its absence of humoral reactivity against axolotl cells, was a long process strictly conducted with this 38-kD eluted material. This succeeded in the obtention of the L12 polyclonal rabbit antiserum, which was then checked for reactivity on axolotl lymphocyte suspensions using indirect simple or double immunofluorescence by optic microscopy and flow cytometry. In all experiments, the L12-0 preimmune serum was negative, which was a good control of the specificity of the L12 reaction. Optic microscopy as well as flow cytometry analysis gave nearly the same results, revealing that L12 or L12 mono-38 antibodies labeled 80–86% of thymocytes and 40–46% of peripheral lymphocytes. This was meaningful and indicated that the surface determinants revealed by the L12 and L12 mono-38 antibodies were expressed by thymocytes and by a subpopulation of splenic lymphocytes. The double immunofluorescence technique with mAb 33.101.2 (anti-axolotl Ig) and L12 or L12 mono-38 antibodies set apart the $mIg^+$ B-cell population and the $L12^+$ splenic lymphocyte subpopulation. A subpopulation of double-positive cells ($L12^+Ig^+$) was detected by flow cytometry (Table 2), as well as by optic microscopy (data not shown). The observation of fluorescent cells with a phase-contrast optic allowed us to distinct morphologically the different cell types involved. In the thymus and the spleen, all the cells labeled with L12 or L12 mono-38 antibodies were typical lymphocytes. The double-positive spleen cells (3–5%) were mostly large granular cells (presumably monocytes and few granulocytes) and probably constituted a significant proportion of the $L12^+Ig^+$ cell detected by flow cytometry. Their labeling could be attributable to the presence of Fc receptors. The double-negative cells ($L12^-Ig^-$) had the same volume as lymphocytes, but with sometimes a more indented nucleus, they could be young erythroblasts or immature lymphocytes (data not shown).

These observations prompt us to characterize further the 38-kD antigen by the use of immunochemical techniques. The Western blotting analysis of the L12 and L12 mono-38 antibodies reactivities on monodimensional SDS-PAGE lysates showed in our first assays that the L12 antiserum was revealing a major single 38-kD band on thymocytes and two major 38- and 36-kD bands on splenocytes. However, in both cases, several other high molecular weight polypeptides were also revealed. No polypeptides were detected by the L12-0 preimmune serum in the same conditions. The additional revelation of high molecular weight proteins was difficult to explain, as the 38-kD immunizing eluted material was systematically checked by Coomassie blue staining for the absence of contaminating material. However, the possibility remained that the long immunization process of the L12 rabbit, in the presence of Freund adjuvant, led to the production of some nonspecific antibodies. When the 38-kD electroeluted material was run a second time in a monodimensional gel and revealed by L12 antibodies after Western blotting, several high molecular weight polypeptides were weakly revealed, beside the 38-kD molecule. The molecular weight calculation of these polypeptides indicated that most of them could represent 38-kD multimers, suggesting that the 38-kD molecule might aggregate spontaneously before or during the course of the SDS-PAGE migration. According to Allore and Barber (1983), some lymphocyte membrane proteins have a tendency to artefactual disulfide bonding in nonreducing conditions. The axolotl lymphocytes were consequently treated with the alkylating reagent iodoacetamide to block secondary disulfide bonding before detergent lysis. Furthermore, strong denaturating conditions and a high 2-mercaptoethanol concentration were used when the lysates were analyzed in reducing conditions. In these new conditions, the 38- and 36-kD proteins were the unique elements revealed by L12 antibodies, demonstrating that the 38-kD polypeptides had a strong spontaneous tendency to associate with themselves, and eventually with other molecules. We also observed that the eluted L12 mono-38 antibodies never revealed any high molecular weight polypeptides even in the absence of alkylation and strong reducing conditions. These antibodies may preferentially recognize some sequential epitopes of the 38-kD molecules that might be obscured by polymer formation or by artefactual bonding with other proteins. However, it was interesting to notice that L12 mono-38 antibodies not only recognized the 38-kD, but also the 36-kD molecules in the spleen. Similarly, L12 mono-36 antibodies recognized the 36-kD as well as the 38-kD species (data not shown). These two antigenically related molecules might be derived from the same precursor. The analysis under nonreducing conditions (Fig. 4, lane 3) suggested that a part of the 36-kD molecules may covalently associate with some yet undefined high molecular weight polypeptide.

The absorption experiments confirmed (1) that the 38- and/or 36-kD proteins are expressed on the lymphocyte surface and (2) that the same antibodies stained the cells in immunofluorescence and recognized 38-kD antigens in immunoblotting.

Our endo-F experiments clearly indicated that the 38-kD electroeluted material behaved heterogeneously after endo-F treatment (Fig. 7). Even after prolonged digestion, a part of the 38-kD material was unaffected and remained further recognizable by L12 antibodies. However, another part of the 38-kD material was endo-F sensitive and gave 36- and 32-kD digestion products that were not recognized further by L12 antibodies. These results could be interpreted in at least two different ways: (1) L12 antibodies recognize carbohydrate determinants and a part of the 38-kD not fully deglycosylated remains recognized by L12; (2) the 38-kD band includes several superposed molecules, a 38-kD polypeptide that is not N-glycosylated and is recognized by L12 and two other polypeptides that are endo-F sensitive and are not recognized (the 36- and 32-kD elements). We favor the second hypothesis in view of our Western blotting experiments after the 2D NEPHGE analysis. The silver nitrate pattern of the spleen and thymus membrane lysates clearly showed numerous high molecular weight spots (Fig. 5). Most of them are probably glycoproteins with their intact carbohydrate determinants not affected by the mild denaturation conditions used in this kind of analysis. If L12 antibodies were specific for carbohydrate determinants, they should stain numerous spots of the 2D patterns, including proteins in the 38–36-kD area. However, this is not the case and the labeling is limited to few spots in a restricted area. Furthermore, these L12-labeled spots do not have the characteristic pattern that is usually seen for glycoproteins.

The two-dimensional profiles in NEPHGE conditions of splenocytes membrane lysates revealed numerous spotted proteins. Among them, the L12 antiserum stained spots organized in some way so that we could conclude to a certain heterogeneity of the 38-kD and 36-kD proteins. But most importantly, thymectomized axolotl displayed very few spots originally recognized by L12. This is a major argument for the specificity of the 38-kD antigen for the T-cell population and confirmed our original observation after monodimensional electrophoresis of thymectomized axolotl splenocyte membranes. The presence of few discrete spots revealed by L12 antibodies in thymectomized axolotl could be explained by the persistence of a small amount of long-lived T cells or by the presence of few thymus-derived cells in the spleen before thymectomy.

Immunoprecipitation experiments using digitonine lysates of $^{125}$I-labeled axolotl splenocytes are now in progress. Our preliminary results indicate that the 38-kD molecules are expressed on the lymphocyte membrane and probably belong to a multimolecular complex.

### MATERIALS AND METHODS

### Animals

Axolotls (*Ambystoma mexicanum*) of the Ax6 black strain were reared at 18–20 °C in our usual laboratory conditions. Thymectomy was performed on 9-month-old larvae as described by Charlemagne and Tournefier (1977).

### Lymphocyte Isolation and Membrane Preparation

Axolotl spleen and thymic nodules were excised and teased with forceps in cold amphibian PBS (A-PBS). Single cell suspensions were prepared in 2% FCS supplemented A-PBS for indirect immunofluorescence analysis.

For membrane preparation and lysates, the single cell suspensions from thymi were used directly and splenic lymphocytes were isolated from other splenocytes by centrifugal sedimentation through discontinuous Ficoll 400 gradient as described previously (Tournefier, 1982). In axolotl, the number of thymocytes never exceed $10 \times 10^6$ and the number of splenocytes $35 \times 10^6$. For that reason, that is, the small number of cells per animal, the classical technique of membrane isolation by ultracentrifugation was not conceivable. A method to obtain crude membrane extracts was adapted from that of Bumstead and Curtis (1986). Thymic and splenic lymphocytes were pelleted and resuspended gently for 10 min in v/v of freshly prepared permabilization buffer consisting of 10% saponin (Sigma) and 0.1-mM phenylmethylsulphonyl fluoride (PMSF) in A-PBS at 4 °C. The cells were then resuspended and washed four times in cold A-PBS. These successive resuspensions and centrifugations allowed to get rid of the cytosol and of most of the cytoplasmic organelles leaking through the saponin permeabilized plasma membrane. The pelleted cells, which at this point, consisted of the nuclei surrounded by the collapsed cell membrane were treated v/v with lysis buffer: 0.5% NP-40, 0.5% deoxycholate (DOC) in 10-mM Tris-HCl, pH 8, 150-mM NaCl, 10-mM $MgCl_2$, 0.1-mM PMSF or 1% digitonin (Sigma) in 20-mM Tris-HCl, pH 8, 150-mM NaCl, 1-mM $MgCl_2$, and 1-mM PMSF, for 1 hr at 4 °C. The lysis buffer was eventually supplemented with 20-mM iodoacetamide (Serva, Heidelberg) according to Allore and Barber (1983). These crude lymphocyte lysates depleted of cytoplasmic components were then centrifuged at 8000 rpm to discard the nuclei and the cytoskeletal debris. The relatively pure membrane lysates, checked by microscopic observation for the total absence of nuclei, were then used for gel electrophoresis and Western blotting analysis.

### Gel Electrophoresis

NP-40/DOC or digitonin lysate samples were analyzed by different electrophoretic techniques.

One-dimensional sodium dodecyl-sulfate polyacrylamide gel electrophoresis (SDS-PAGE) was carried out on vertical 12.5% gels according to Laemmli (1970), or 8–16% gradient gels under reducing (R) and nonreducing (NR) conditions. Approximately 20 $\mu$g of membrane lysate protein were loaded in individual lanes as estimated according to the Bradford (1976) technique.

Two-dimensional nonequilibrated pH electrophoresis (NEPHGE) was also used. The first-dimension gels were done on 4% polyacrylamide containing 2% ampholines (Pharmacia, Uppsala, Sweden), pH 3.5–10, 5–8, (4:1), to resolve basic proteins. The second dimension was carried out on 12.5% polyacrylamide gels. Particular care was taken to load identical amounts of membrane lysates (equivalent in cell number) in the analytical gels.

### Electroelution

For protein elution, the polyacrylamide gels were fixed and stained with Coomassie blue G-250 (Serva) to identify the band of interest. The appropriate area was cut out and the 38-kD protein electroeluted at 20 mA constant current for 3 to 4 hr with a 422 Biorad eluter (Biorad, France).

After each electroelution, a sample of the electroeluted product was secondarily electrophoresed on 12.5% gradient SDS-PAGE gel and controlled after staining with Coomassie blue. In all cases, the electroeluted material was demonstrated to be exclusively composed of the 38-kD polypeptide.

### Protein Deglycosylation

The 38-kD electroeluted protein was incubated at 37 °C either with or without endo-$\beta$-N-acetylglucosaminidase F (endo-F) from *Flavobacterium meningosepticum* (Boehringer, Mannheim) in a buffer containing 0.1-M sodium phosphate, pH 6.1, 0.5% NP-40, 0.1% SDS, 20-mM EDTA. Different concentrations from 0.1 to 0.25 U of endo-F

were used as well as different incubation times: 2, 4, 6, and 18 hr for each concentration. The deglycosylated samples were denatured by boiling for 3 min in 0.0625-M Tris-HCl, pH 6.8, 2% SDS, 5% 2-mercaptoethanol, and 10% glycerol.

### Antibodies

*Rabbit antiserum against the 38-kD molecule.*

Among 10 rabbits (New Zealand) tested prior immunization for their serum reactivity in Western blotting on axolotl membrane proteins, and in indirect immunofluorescence on splenic axolotl lymphocytes, one rabbit (L12) was selected for its complete absence of reactivity with axolotl lymphocytes. The preimmune serum of this rabbit (L12-0), obtained by partial ear bleeding prior to immunization, was used systematically as negative control in all the described experiments. The immunization protocol was a long process: A first i.d. foot pad injection of a mixture v/v of the electroeluted 38-kD protein in CFA was followed by three s.c. injections of the same antigen in IFA at 1-month intervals. Three months later, two booster i.v. injections of an alum precipitate of the same 38-kD protein were performed at 2-week intervals. This immunization was completed 1 month later by a last s.c. injection of the same antigen in IFA. The rabbit (L12) was finally bled 10 days after the last injection. The polyclonal L12 antiserum was purified on DEAE-Trisacryl M according to Saint-Blancard et al. (1982) and used as such for immunoblotting, and flow cytometry.

*Rabbit L12 mono-38.*

The rabbit polyclonal antibodies were concentrated and rendered monospecific (L12 mono-38) for the 38-kD membrane protein by absorption on a nitrocellulose band blotted exclusively with the isolated and electroeluted 38-kD molecule. Briefly, the electroeluted 38-kD molecule was electrophoresed in one-dimensional 12.5% SDS-PAGE and blotted onto a nitrocellulose sheet. The two extremities of the sheet were cut out and immunostained with the L12 antiserum by Western blotting, to ascertain that the 38-kD molecule was in the correct molecular weight range. The nitrocellulose band thus located, and containing the 38-kD molecule, was then cut out of the remaining nitrocellulose sheet and used as absorbant. The absorbed mono-38 antibodies were eluted from the nitrocellulose with 3M KSCN, pH 6.4, and concentrated according to Olmsted (1981) modified by Krohne et al. (1982).

*Monoclonal and conjugated antibodies.*

MAb 33.101.2 (mouse IgG1$\kappa$) raised against axolotl Ig light chains was obtained and purified as described by Tournefier et al. (1988a). FITC conjugated anti-rabbit Ig was purchased from Dakopatts (Glostrup, Denmark) and biotinylated anti-mouse Ig and PE-streptavidin from Amersham (Amersham Ltd., Amersham, UK).

### Western Blotting

Proteins separated by 1D or 2D SDS-PAGE were electrophoretically transferred onto nitrocellulose and revealed by incubation with L12-0, L12, or L12 mono-38 antibodies, followed by peroxydase-conjugated swine anti-rabbit IgG (Dakopatts), as previously described (Guillet et al., 1990).

### Immunofluorescence Staining and Flow Cytometry

Cells were stained with polyclonal L12 or L12 mono-38 antibodies followed by FITC-conjugated anti-rabbit Ig or with mAb 33.101.2 revealed by biotinylated anti-mouse Ig followed by PE-streptavidin. Alternatively, cells were doubly stained by (1) mAb 33.101.2 followed by biotinylated anti-mouse Ig revealed by PE-conjugated streptavidin and (2) by L12 antibodies followed by FITC-conjugated swine anti-rabbit Ig. Before (2), cells were treated with mouse Ig at 1 mg/ml to block cross-reactive antibody sites. Labeled cells were examined visually with a Leitz Orthoplan microscope equipped with a Ploem illuminator, phase-contrast optics, and appropriate filters. At least 500 lymphocytes were numbered. Cells were also analyzed with a flow cytometer ACR 1400 ST (Brucker Spectrospin, Wissembourg, France) with logarithmic intensity scales. The gated fluorescence histograms were acquired on $5 \times 10^3$–$10^4$ lymphocytes. Dead cells were gated out with a combination of forward and right-angle light scatter.

### ACKNOWLEDGMENTS

We thank Dr. L. Du Pasquier of the Basel Institute for Immunology for helpful discussions and advice. We are grateful to Dr. E. Baviera, C. Gastal for assistance with flow cytometry and to Dr. D. Boucher for help with the two-dimensional technique, A. M. Dumay and G. Aubet for technical cooperation, B. Cuvelier and J. Desrosiers for typing and illustrations. This work was supported by Université Pierre et Marie Curie and Centre National de la Recherche Scientifique (URA 1135).

*(Received April 24, 1991)*

*(Accepted November 19, 1991)*

### REFERENCES

Allore R.J., and Barber B.H. (1983). Inter- and intramolecular disulfide bonding among lymphocyte plasma membrane proteins and glycoproteins. Mol. Immunol. **4:** 393–395.

Bradford M.M. (1976). A rapid and sensitive method for the quantitation of microgram quantities of proteins utilizing the principle of protein-dye binding. Anal. Biochem. **72:** 248–254.

Bumstead N., and Curtis N.J. (1986). Identification of chicken major histocompatible complex (B) haplotypes by an immunoblot techniques. J. Immunol. Meth. **95:** 267–271.

Chardin H., Vilain C., and Charlemagne J. (1987). Characterization of axolotl heavy and light immunoglobulin chains by monoclonal antibodies. Hybridoma **6:** 627–635.

Charlemagne J. (1983). Effects of immunization, adult thymectomy and irradiation on axolotl spleen lymphocytes: Discontinuous Ficoll density gradients analysis. Dev. Comp. Immunol. **7:** 385–389.

Charlemagne J. (1990). Immunologie des poïkilothermes. *In: Immunologie Animale,* Pastoret P.P., Gewaerts A., and Bazin H. Eds. (Paris: Médecine-Sciences Flammarion), pp. 447–457.

Charlemagne J., and Tournefier A. (1977). Humoral response to *Salmonella typhimurium* antigens in normal and thymectomized urodele amphibian *Pleurodeles waltlii* Michah. Eur. J. Immunol. **7:** 500–502.

Ching Y.C., and Wedgwood R.J. (1967). Immunologic response in the axolotl, *Siredon mexicanum.* J. Immunol. **99:** 191–200.

Du Pasquier L. (1989). The immune system of the *Xenopus.* Ann. Rev. Immunol. **7:** 251–275.

Du Pasquier L., and Haimowitch J. (1976). The antibody response during amphibian ontogeny. Immunogenetics **3:** 381–391.

Guillet F., Tournefier A., Denoulet P., Capony J.P., Kerfourn F., and Charlemagne J. (1990). High levels of HMG1-2 protein expression in the cytoplasm and nucleus of hydrocortisone sensitive amphibian thymocytes. Biol. Cell. **69:** 153–160.

Houdayer M., and Fougeréau M. (1972). Phylogénie des immunoglobulines: La réaction immunitaire de l'axolotl "*Ambystoma mexicanum.*" Cinétique de la réponse immunitaire et caractérisation des anticorps. Ann. Immunol. (Institut Pasteur). **123:** 3–28.

Krohne G., Sthick R., Kleinschmidt J.A., Moll R., Franke W.W., and Hausen P. (1982). Immunological localization of a major karyoskeletal protein in nucleoli of oocytes and somatic cells of *Xenopus laevis.* J. Cell. Biol. **94:** 749–754.

Laemmli U.K. (1970). Cleavage of structural proteins during the assembly of the head of bacteriophage T4. Nature (London) **227:** 680–685.

Litman G.W. (1976). Physical properties of immunoglobulins of lower species: A comparison with immunoglobulins of Mammals. *In: Comparative Immunology,* Marchalonis, J.J., Ed. (Oxford: Blackwell), p. 239.

Litman G.W., and Marchalonis J.J. (1982). Evolution of antibodies. *In: Biological Significant of Immune Regulation,* Gershwin E., and Ruben, L.N. Eds. (New York: Marcel Dekker), pp. 29–60.

Lobb C.J., and Clem L.W. (1982). Fish lymphocytes differ in the expression of surface immunoglobulin. Dev. Comp. Immunol. **6:** 473–479.

Miller N.W., Sizemore R.C., and Clem L.W. (1985). Phylogeny of lymphocyte heterogeneity: The cellular requirements for *in vitro* antibody responses of channel catfish leukocytes. J. Immunol. **134:** 2884–2888.

Miller N.W., Bly J.E., van Ginkel F., Elsaesser C.F., and Clem L.W. (1987). Phylogeny of lymphocyte heterogeneity: Identification and separation of functionally distinct subpopulations of channel catfish lymphocytes with monoclonal antibodies. Dev. Comp. Immunol. **11:** 739–747.

Nagata S. (1985). A cell surface marker of thymus-dependent lymphocytes in *Xenopus laevis* is identifiable by mouse monoclonal antibody. Eur. J. Immunol. **15:** 837–841.

Nagata S. (1988). T cell specific antigen in *Xenopus* identified with a mouse monoclonal antibody: Biochemical characterization and species distribution. Zool. Sci. **5:** 77–83.

Nagata S., and Katagiri C. (1978). Lymphocyte surface immunoglobulin in *Xenopus laevis.* Light and electron microscopic demonstration by immunoperoxidase method. Dev. Comp. Immunol. **2:** 277–286.

Olmsted J.B. (1981). Affinity purification of antibodies from diazoted paper-blots of heterogenous proteins samples. J. Biol. Chem. **256:** 11955–11957.

Saint-Blancard J., Foucart J., Girot P., and Boschetti E. (1982). Préparation de l'albumine et des IgG par fractionnement chromatographique direct du plasma humain sur DEAE et CM-TRISACRYL M. Revue Française de Transfusion et d'Immuno-hématologie **25:** 1.

Secombes C.J., Van Groningen J.J.M., and Egberts E. (1983). Separation of lymphocyte subpopulations in carp *Cyprinus carpio* L. by monoclonal antibodies: Immunohistochemical studies. Immunology **48:** 165–175.

Tomonaga S., and Kobayashi K. (1985). A second class of immunoglobulin in the cartilaginous fishes. Dev. Comp. Immunol. **9:** 797–802.

Tournefier A. (1982). Corticosteroid action on lymphocyte subpopulations and humoral immune response of axolotl (urodele amphibian). Immunology **46:** 155–162.

Tournefier A., and Guillet F. (1987). Monoclonal antibody distinguishes hematopoietic precursor cells and leucocytes in the axolotl: Ontogenesis study. Dev. Comp. Immunol. **11:** 453.

Tournefier A., Fellah S., and Charlemagne J. (1988a). Monoclonal antibodies to axolotl immunoglobulins specific for different heavy chains isotypes expressed by independent lymphocyte subpopulations. Immunol. Lett. **18:** 145–148.

Tournefier A., Guillet F., Ardavin C., and Charlemagne J. (1988b). Surface markers of axolotl lymphocytes as defined by monoclonal antibodies. Immunology **63:** 269–276.

